# Arginine Vasotocin, the Social Neuropeptide of Amphibians and Reptiles

**DOI:** 10.3389/fendo.2017.00186

**Published:** 2017-08-07

**Authors:** Walter Wilczynski, Maricel Quispe, Matías I. Muñoz, Mario Penna

**Affiliations:** ^1^Neuroscience Institute, Center for Behavioral Neuroscience, Georgia State University, Atlanta, GA, United States; ^2^Programa de Fisiología y Biofísica, Instituto de Ciencias Biomédicas, Facultad de Medicina, Universidad de Chile, Santiago, Chile

**Keywords:** vasotocin, urodeles, anurans, amphibians, reptiles, communication, aggression

## Abstract

Arginine vasotocin (AVT) is the non-mammalian homolog of arginine vasopressin (AVP) and, like vasopressin, serves as an important modulator of social behavior in addition to its peripheral functions related to osmoregulation, reproductive physiology, and stress hormone release. In amphibians and reptiles, the neuroanatomical organization of brain AVT cells and fibers broadly resembles that seen in mammals and other taxa. Both parvocellular and magnocellular AVT-containing neurons are present in multiple populations located mainly in the basal forebrain from the accumbens–amygdala area to the preoptic area and hypothalamus, from which originate widespread fiber connections spanning the brain with a particularly heavy innervation of areas associated with social behavior and decision-making. As for mammalian AVP, AVT is present in greater amounts in males in many brain areas, and its presence varies seasonally, with hormonal state, and in males with differing social status. AVT’s social influence is also conserved across herpetological taxa, with significant effects on social signaling and aggression, and, based on the very small number of studies investigating more complex social behaviors in amphibians and reptiles, AVT may also modulate parental care and social bonding when it is present in these vertebrates. Within this conserved pattern, however, both AVT anatomy and social behavior effects vary significantly across species. Accounting for this diversity represents a challenge to understanding the mechanisms by which AVT exerts its behavioral effects, as well are a potential tool for discerning the structure-function relationships underlying AVT’s many effects on behavior.

## Introduction

**Figure 1:**
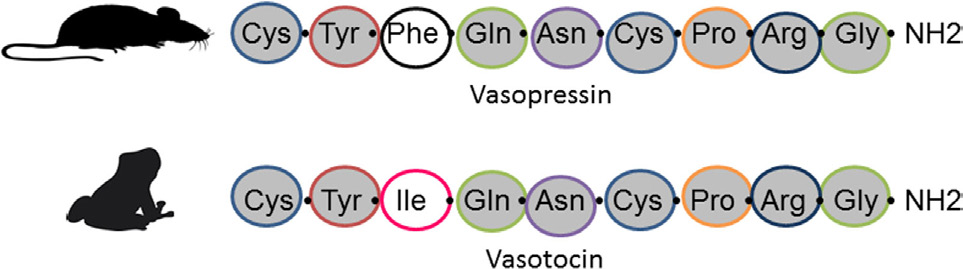
Amino acid sequence in vasopressin (top) found in mammals and arginine vasotocin (bottom) found in amphibians, reptiles, and other non-mammalian vertebrates.

Mammalian vasopressin is one of a small group of nine-amino acid peptides whose wide and consistent distribution across animals indicates a long phylogenetic history (1). Both the peptides and their receptors are highly conserved in their structure and expression in neural and non-neural tissue (2, 3). Whereas invertebrates have a single nonapeptide differing slightly among taxonomic groups, vertebrates have two such peptides. In mammals, these are vasopressin and oxytocin, and other vertebrates have two homologous peptides with similar structures. Amphibians and reptiles, like other non-mammalian vertebrates, express arginine vasotocin (AVT) rather than the arginine vasopressin (AVP) found in mammals. AVT differs from AVP by a single amino acid (Figure 1).

Despite vast differences in overall nervous system structure and in species-specific behavioral repertoires and physiology, vasopressin, and its peptide homologs demonstrate a remarkable conservation in their function. In all cases, peripheral physiological effects target osmoregulation and smooth muscle contraction, particularly in reproductive organs. In the nervous system, they have profound impacts on social behavior. Most importantly for behavioral neuroendocrinology, the AVT/AVP nonapeptide and its invertebrate homologs invariably modulate social communication, reproduction, and aggression. Amphibians and reptiles share this common feature with all other organisms along with a similar neuroanatomical distribution of AVT cells and fibers.

## Anatomy of the AVT System in Amphibians and Reptiles

The locations of AVT/AVP cells have been described in all groups of vertebrates, including cartilaginous and bony fish, amphibians, reptiles, birds, and mammals. Although there are variations in the extent and position of neuronal populations expressing this peptide, the general anatomical organization of AVT/AVP populations and fibers is conserved across vertebrates (4, 5). Several magnocellular and parvocellular cell groups are located in the basal forebrain extending from the amygdala/nucleus accumbens (NAcc) through the preoptic area (POA) to the hypothalamus. These give rise to a neurosecretory pathway to the posterior pituitary as well as an extensive AVT fiber system throughout the central nervous system with particularly heavy innervation of the limbic system. Anatomical abbreviations used in text, tables and figures are found in the abbreviation section; Table 1 summarizes the locations of AVT cells across amphibian and reptilian species.

**Table 1:** Brain regions containing arginine vasotocin (AVT) cells in different amphibian and reptile species.

Group	Species	Family	Dp	Mp	Lp	Ms	Ls	NAcc	Str	Ma	BNST	POA/PVN-SON	SCN	AH	VM/VL	Midbrain	Hindbrain	Reference
**Amphibians**																		
Urodela (newts and salamanders)	*Pleurodeles waltl*	Salamandridae									x	x					x (1)	González and Smeets (6)
	*Taricha granulosa*	Salamandridae	x	x		x				x	x	x			x	x (2) (3)	x (1) (4)	Lowry et al. (7)
	*Plethodon shermani*	Plethodontidae	x	x	x	x				x	x	x				x (5)	x (1) (6)	Hollis et al. (8)
	*T. granulosa*	Salamandridae	x	x		x	x			x	x	x			x	x (5) (2)	x (1) (6)	Hollis et al. (8)
Anura (toads ad frogs)	*Rana temporaria*	Ranidae										x						Vandesande and Dierickx (9)
	*Rana esculenta* (=*Pelophylax bedriagae*)	Ranidae										x						Vandesande and Dierickx (9)
	*Bubo bufo*	Bufonidae										x						Vandesande and Dierickx (9)
	*Bubo japonicus*	Bufonidae										x						Jokura and Urano (10, 11)
	*Xenopus laevis*	Pipidae								x	x	x	x		x	x (7)		González and Smeets (6, 12)
	*Rana ridibunda* (=*Pelophylax ridibundus*)	Ranidae						x	x	x	x	x	x		x	x (3)	x (8)	González and Smeets (6)
	*Rana catesbeiana* (=*Lithobates catesbeianus*)	Ranidae				x		x	x	x	x	x	x				x (9)	Boyd et al. (13)
	*Acris crepitans*	Hylidae						x	x	x		x	x					Marler et al. (14)
	*Hyla cinerea*	Hylidae						x		x		x						O’Bryant and Wilczynski (15)
	*H. cinerea*	Hylidae						x	x	x		x	x		x			Lutterschmidt and Wilczynski (16)
	*H. cinerea*	Hylidae						x	x	x		x	x		x			Howard and Lutterschmidt (17)

**Reptiles**																		
Squamata (lizards)	*Lacerta muralis*	Lacertidae										x						Bons (18)
	*Acanthodactylus paradis*	Lacertidae										x						Bons (18)
	*Acanthodactylus boskianus*	Lacertidae										x						Bons (18)
	*Tarentola mauritanica*	Gekkonidae										x						Bons (18)
	*Gekko gecko*	Gekkonidae									x	x					x(10)	Stoll and Voorn (19)
	*G. gecko*	Gekkonidae									x	x					x(10)	Thepen et al. (20)
	*Anolis carolinensis*	Dactyloidae				x						x		x	x	x (3)		Propper et al. (21, 22)
	*A. carolinensis*	Dactyloidae										x		x				Hattori and Wilczynski (23)
	*Anolis sagrei*	Dactyloidae									x	x						Kabelik et al. (24)
	*Cnemidophorus uniparens (*=*Aspidoscelis uniparens)*	Teiidae									x	x		x				Hillsman et al. (25)
	*Urosaurus ornatus*	Phrynosomatidae										x	x					Kabelik et al. (26)
Squamata (snakes)	*Natrix maura*	Natricidae										x						Fernández-Llebrez et al. (27)
	*Python regius*	Pythonidae									x	x						Smeets et al. (28)
	*Bothrops jararaca*	Viperidae										x						Silveira et al. (29)
Testudines (turtles)	*Pseudemys scripta (*=*Trachemys scripta)*	Emydidae									x	x						Smeets et al. (28)
	*Mauremys caspica*	Geoemydidae										x						Fernández-Llebrez et al. (27)

*The presence of AVT cells is indicated by x under the corresponding nuclei, which are listed in rostrocaudal order. POA includes the entire preoptic area for amphibians. We combined POA with the more caudal nuclei the PVN and SON in a single column, as reports in amphibians do not generally distinguish separate POA/PVN/SON areas; and whereas recent work on lizards indicate AVT-immunoreactive cells to be in distinctly separate PVN and SON nuclei, earlier studies in reptiles do not always clearly make that distinction. In the midbrain and hindbrain columns, the numbers in parentheses indicate specific regions in which AVT cells are found: (1) nucleus isthmi, (2) torus semicircularis (inferior colliculus), (3) interpeduncularis nucleus, (4) lateral auricle area, (5) optic tectum, (6) eminentia trigemini, (7) an area dorsolateral to oculomotor nucleus, (8) nucleus of the solitary tract, (9) pretrigeminal nucleus, (10) nucleus reticularis. We emphasize that the lack of reported AVT cells and fibers should be considered very cautiously, as it can be due to any number of technical differences across species and labs and could reflect the variable nature of AVT densities related to particular social or seasonal conditions (see Anatomical Variation by Group, Sex, and Season) rather than a strict species trait, or simply that the particular published study did not investigate that area. See abbreviation section for abbreviations used*.

### AVT in Urodeles (Salamanders and Newts)

Arginine vasotocin has a widespread distribution from forebrain to hindbrain in salamanders and newts [for reviews see Ref. (30, 31)]. We employ the nomenclature of Gonzalez and Smeets in this article. In all three urodele species (*Taricha granulosa, Pleurodeles waltl*, and *Plethodon shermani*) for which brain AVT distribution has been studied to date (7, 8, 12) AVT cells occur in the bed nucleus of the stria terminalis (BNST) and POA. In addition, in *T. granulosa* and *P. shermanii* AVT cell populations have been identified in pallium [dorsal pallium and medial pallium (Mp)], and subpallial limbic areas [medial septum (Ms) and medial amygdala (MA)], the suprachiasmatic nucleus (SCN), ventral thalamus, nucleus isthmi, optic tectum, and torus semicircularis (inferior colliculus). In addition, in *P. waltl* and *T. granulosa*, AVT neurons have been identified in the ventromedial hypothalamus (7, 12). A feature common to several of these nuclei is their involvement in the social decision-making network, a group of brain nuclei that modulate behaviors related to socially salient stimuli (32).

Arginine vasotocin cells from these nuclei send projections to many different brain regions forming an extensive network of fibers. In *T. granulosa* and *P. waltl* (7, 12) fibers are present from the olfactory bulb to the cervical segments of the spinal cord. AVT fiber density is not homogeneous throughout the brain. Denser fiber aggregations of variable thickness are found mainly in the vicinity of the lateral pallium, Mp, and POA.

### AVT in Anurans (Frogs and Toads)

**Figure 2:**
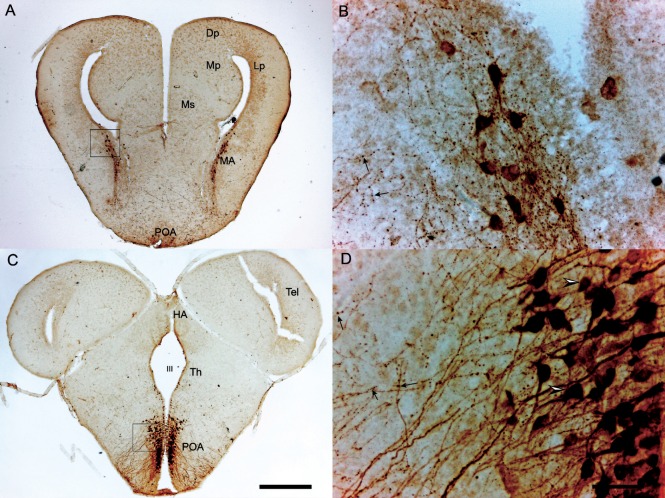
Transverse sections through the brain of a *Pleurodema thaul* male showing arginine vasotocin (AVT)-immunoreactive cells at the level of amygdala **(A,B)** and preoptic area (POA) **(C,D)**. **(A)** AVT cells in the medial amygdala (MA). AVT cells in the inset are magnified in **(B)**; arrows indicate axonal varicosities characteristic of AVT neurons in many vertebrates. **(C)** AVT cells in the caudal POA. AVT cells in the inset are magnified in **(D)**; arrowheads show parvo- (upper) and magnocellular (lower) neurons, which are interspersed in this magnocellular region of the POA. Scale bars on panel **(C)** (0.5 mm) and **(D)** (50 µm) also apply to **(A,B)** respectively. See abbreviation section for abbreviations used.

A larger number of studies have investigated the presence of AVT cells in the brain of anurans as compared to urodeles and includes descriptions in more species from different families (6, 9–14, 16, 17). Comprehensive whole-brain AVT immunocytochemical studies have been conducted in five species: *Xenopus laevis, Rana catesbeiana* (=*Lithobates catesbeianus*), *Rana ridibunda* (=*Pelophylax ridibundus*), *Acris crepitans*, and *Hyla cinera*. In all five species, AVT cells are located in the MA, POA, SCN, and VM. In four of the species listed above AVT cells also occur in the striatum and NAcc, but this was not reported in *X. laevis* (6). Figure 2 shows AVT cells in the MA and POA of the South American frog *Pleurodema thaul*. For simplicity, we refer to the entire region as the POA, as finer distinctions are often not made in amphibian neuroanatomy papers. However, when subregions are identified, the amphibian POA is usually divided along the rostral–caudal axis into anterior, magnocellular, and posterior areas. Most magnocellular AVT-containing neurons with projections apparently going to the median eminence are located in the magnocellar division of the POA where they are interspersed with AVT-positive parvocellular neurons. Some magncellular neurons are also located in other parts of the POA. We assume that the magnocellular POA is equivalent at least in part to the PVN of other tetrapods, but published reports have generally not identified it as such. AVT cells are also present in midbrain and hindbrain nuclei of some of these species (Table 1); however, AVT cells at these levels are not as abundant as in forebrain regions (6, 12, 13). In *L. catesbeianus*, AVT cells are also found in the Ms; this frog species has the largest number of AVT nuclei identified to date (13). A thorough comparative analysis of anuran AVT cell populations that takes into account connections, cell morphology, and molecular markers is needed to clarify AVT cell group homologies in the amphibian POA and other areas so that they can be compared with those in other vertebrates.

As in urodeles, AVT fibers occur in regions along the entire anuran brain, from olfactory bulb to spinal cord (6, 12, 13). The POA contains the most dense plexus of AVT fibers, apparently originating in AVT cells located in this same nucleus. Other extra-hypothalamic regions with an ample distribution of AVT fibers are Ms, NAcc, BNST, and MA in the forebrain and also the optic tectum, torus semicircularis, tegmentum, and pretrigeminal nucleus at the midbrain and hindbrain levels. The torus, tegmentum, and pretrigeminal nucleus are regions that participate in reception or production of calls by frogs (33, 34), and therefore, even though these nuclei generally lack AVT cells, the presence of AVT fibers could be relevant for AVT effects on frog vocal behavior (see Effects of AVT on Male Reproductive Behavior).

### AVT in Reptiles

The reptile AVT system has been described in lizards (18–26), turtles (27), and snakes (27–29). The supraoptic nucleus (SON) and PVN are the most conspicuous AVT nuclei in reptiles and are considered homologous to the AVT-containing magnocellular POA in amphibians (30). All reptile species investigated to date have AVT cells in these nuclei. It is noteworthy that, unlike in anurans, AVT cells have not been reported in NAcc or MA in reptiles.

In all lizards so far examined, with the exception of *Gekko gecko* (19, 20), numerous AVT cells also occur in the POA rostral to the area identified as equivalent to the mammalian SON plus PVN (18, 21, 23–26). This area is sometimes termed the anterior hypothalamus (21, 23, 25). Smaller groups of AVT cells have been reported in the BNST (19, 20, 24, 25). AVT cells have also been reported in the hindbrain in *G. gecko* (19, 20), and another unique case is the occurrence of AVT cells in the Ms and interpeduncularis nucleus in *Anolis carolinensis* (22).

In comparison to lizards, turtles and snakes have fewer AVT-containing nuclei, and only the SON and PVN have been reported to have the neuropeptide in the turtle *Mauremys caspica* and in the snakes *Natrix maura* and *Bothrops jararaca* (27, 29). The BNST also contains AVT cells in the turtle *Pseudemys scripta elegans* and the snake *Python regius* (28).

The distribution of AVT fibers in the brains of different reptiles is comparable to amphibians in its extent. Detailed studies in lizards (19, 20, 22, 26) as well as in turtles and snakes (27–29) report AVT fibers extending from forebrain to spinal cord, passing through olfactory bulb, lateral septum (Ls), Dc, NAcc, MA, periaquaductal gray (PAG), and locus coeruleus.

### Anatomical Variation by Group, Sex, and Season

Although AVT cells have been reported to occur in multiple nuclei within the urodele, anuran, and reptile central nervous systems, the reported areas do vary. For example, in frogs, but not urodeles or reptiles, AVT cells are present in the NAcc, and AVT cells are present in the cortex of reptiles and homologous pallium in urodeles, but not in anurans. Because the reports span many years and many different labs, often using slightly different nomenclature, it is difficult to reconcile these reports, and as always the reported absence in an area must be considered with caution. Despite these difficulties, important common patterns are apparent across amphibians and reptiles, and between these herpetological groups and mammals. Both magnocellular and parvocellular AVT neurons are found, with the former indicated as the neurosecretory AVT cells. The magnocellular cells are found in a discrete population mixed with parvocellular cells in the POA in a region called the amphibian POA (Figure 2) or sometimes the magnocellular POA to distinguish it from the more rostral anterior POA. Distinct PVN and SON nuclei with AVT-immunoreactive (AVT-ir) cells are now recognized with a more distinctly separate magnocellular population in the PVN [e.g., see Ref. (26)]. Parvocellular AVT populations are found in multiple regions of the hypothalamus and forebrain limbic system and in all species an extensive AVT fiber system extends throughout the brain. A clearer understanding of the homologous relationships of AVT/AVP populations across vertebrates awaits additional comparative work, and the different functions each cell group may have in regulating social behavior or other processes needs further investigation as well.

Sex differences in immunoreactive cell number or fiber density are apparent in amphibians and reptiles. Usually these favor males, but in some areas females show more AVT. Relative to females, *T. granulosa* males have more AVT cells in the POA, MA, and BNST (13, 35) and higher AVT concentrations in the optic tectum and tegmentum (36). Remarkably, during the breeding season, *T. granulosa* females possess a higher number of AVT cells in the hypothalamus relative to males, but out of the breeding season, no such difference is expressed (36). Male-biased sexual dimorphisms also occur in frogs (13, 14, 16, 35). For example, male bullfrogs have a higher number of AVT cells and denser or more numerous AVT fibers in the MA and habenula; in contrast, females have larger AVT cells than males in the SCN (13). Boyd and Moore (35) reported AVT concentrations, as measured with radioimmunoassay, to be higher in males than in females in the La, optic tectum, and tegmentum, but in the dorsolateral nucleus of the mesencephalon AVT concentration was higher in females. Less work on AVT sex differences has been done in reptiles, but males have been reported to have a higher density of AVT fibers than females, particularly in the PAG and Ls (19, 20, 28), Dc (22), and in the amygdala, BNST, NAcc, and POA in addition to the Ls (26) in a variety of species.

Arginine vasotocin patterns also vary seasonally and with sex steroid hormone levels in all three groups. In *T. granulosa*, higher concentrations of AVT are found in the optic tectum during the breeding season than out of it, whereas in the dorsal POA AVT concentrations are higher out of the mating period (37). Boyd et al. (13) reported evidence for AVT seasonal variation in bullfrogs, with the vocal premotor pretrigeminal nucleus expressing AVT-ir cells only during the fall. Gonadectomy in bullfrog males and females decreases AVT concentration in most of the nuclei containing AVT cells, and treatment with gonadal steroids increases it (38). Seasonal differences in AVT fiber density have been found in the tree lizard (*Urosaurus ornatus*), with higher densities in peak and late breeding seasons compared to early in the season (26). There are relatively few studies of hormonal effects. Kabelik et al. (26) reported that testosterone implants in males increased soma size in both magnocellular and parvocellular AVT cells as well as higher fiber densities in the PVN and more rostral limbic areas. Whiptail lizards (*Cnemidophorus uniparens* and *C. inornatus*) treated with testosterone implants have a larger number of AVT neurons in the POA, but not other brain areas, compared with animals castrated and with blank implants (25). However, a correlation between AVT cell number and naturally circulating testosterone levels is not apparent in green anoles (*A. carolinensis*) (23). More work on reptiles in this area is needed, both for identifying conserved regulatory features impacting AVT across vertebrates, and for supporting the behavioral endocrinology work on lizard aggression and social hierarchy formation.

## Effects of Vasotocin on Behavior in Urodeles

Investigations of AVT’s modulation of social behavior in herpetological taxa started with work by Diakow on release calls in frogs (reviewed in Section “Effects of AVT on Female Reproductive Behavior”) and expanded with Frank Moore’s extensive research program that established salamanders as a model organism for AVT research [reviewed in Ref. (39, 40)]. Most of the now classic research in salamanders has focused on an early phase of courtship behavior, namely the clasp response of males on females during which the male positions himself on the female’s back and tightly grasps her around her pectoral region, a behavior called “amplexus.” Amplexus is an important component of courtship, especially in species with internal fertilization, comprising above 90% of urodeles (41).

More recent studies have explored AVT’s modulation of signaling and sensory capabilities relevant for breeding interactions between males and females. Salamanders and newts have evolved chemical communication as an important mediator of courtship and mating behavior (42). Visual stimuli are also important, and tactile stimulation operates during courtship contact episodes between males and females (40, 43, 44). All are targets of AVT’s modulation.

### Effects of AVT on Male Courtship Behavior

Most of the studies of AVT’s effects on male urodeles has been conducted in *T. granulosa*, from the family Salmandridae. This species has internal fertilization, and during courtship males pursue female to amplex them. Intraperitoneally injected AVT stimulates amplextic clasping in males exposed to receptive females (45). Moore and Miller (46) reported the same effect in males that received intracerebroventricular (ICV) injections of the neuropeptide; the effect on clasping behavior was stronger and the doses required were lower. Furthermore, ICV injections of the AVT receptor antagonist Manning Compound reduced clasping. This early study (46) and a later one by Toyoda et al. in the Japanese newt *Cynops pyrrhogaster* (47) are the only direct evidence to date in this species or any other amphibian or reptile that AVT can exert its action at the central nervous system level rather than depending solely on a peripheral, systemic action as proposed previously in early studies on the AVT stimulation of anuran release calls (48).

In addition to modulation of the clasping response, AVT influences male responses to and production of sexual signals. Male *T. granulosa* show more approaches and time in proximity to models scented with female sex pheromones after intraperitoneal AVT injections, relative to saline injected controls (43). AVT also affects the production of other types of courtship signals. The Japanese newt *C. pyrrhogaster* uses tail vibrations during courtship that occur concomitant with release of sodefrine from the male abdominal gland. Both displays are enhanced in AVT-injected males relative to controls, both in systemic and ICV administration and in a dose-dependent manner (47). The deposition of the male spermatophore is also activated in a dose-dependent manner. Recent studies have been conducted on another Asian salamander *Hynobious leechi*, an external fertilization breeder that does not perform amplexus. Males in this species produce body undulations which generate water waves that stimulate egg laying by conspecific females. AVT-injected *H. leechi* males show higher undulation rates both during the breeding season and out of the breeding season, relative to controls (49). Interestingly, AVT-injected animals show larger undulation rates in the presence of female scent than unexposed animals, indicating AVT modulation of olfactory sensitivity as well as signal production in this species. In both species, treatment with vasopressin V1 receptor antagonists reduced the behavior (47).

Early work related AVT levels in various brain regions to behavioral states as indirect evidence for AVT’s role in urodele social behavior (50, 51). However, many more neural studies conducted in this group of amphibians have emphasized neurophysiological approaches focusing on motor and sensorimotor areas of the brainstem where AVT fibers terminate (40, 52). This emphasis arises from the tradition of investigating AVT modulation of male clasping in this amphibian group, that is, specifically on the motor component of courtship. In *T. granulosa* males, neurons in the rostral medulla reticular formation respond to cloacal pressure, a signal relevant for clasping behavior. Neurons there increase their spontaneous firing as well as their response to clasp-triggering cloacal stimulation after males are injected with AVT (53). This suggests that modulation of the sensitivity of this group of neurons by the neuropeptide could be relevant for AVT’s stimulation of clasping behavior.

Arginine vasotocin innervation of sensory areas is also robust, reaching multiple olfactory areas including the olfactory bulb and midbrain visual areas (52). In many sensory areas, AVT fiber density is sexually dimorphic and higher in males (35). Both modalities are important in male mate searching, and AVT treatment enhances responses to female pheromones as well as broadly increases responses to visual stimuli (43, 44).

### Effects of AVT on Female Behavior

In contrast to the body of work on AVT effects in male urodeles, there has been little investigation of its modulation of female behavior. AVT treatment does elicit oviposition in female *T. granulosa* (54). However, it is not clear whether this is simply a result of AVT stimulation of oviduct contraction, a well-known phenomenon in multiple amphibians and reptiles (55). It also increases a female-typical clasping behavior that in nature is used to attach the inseminated eggs to underwater foliage and other objects. AVT has no effect in ovariectomized females unless they are subsequently treated with estrogen, indicating that AVT alone is not sufficient to trigger this behavior. Interestingly, implanting ovariectomized females with dihydrotestosterone shifts female clasping preferences from foliage to other females (54). It is not at all clear how “male-like” this female-to-female clasping is. However, the work is instructive in showing that the female’s steroid environment interacts with AVT to direct the target of the individual’s response. A similar interaction related to olfactory investigation was described by Thompson and Moore (44). Female *T. granulosa* do not approach female scented models as do males, and AVT alone does not influence such olfactory or visual sexual exploratory behavior in intact or estrogen-implanted females. However, females that were ovariectomized and received testosterone implants and an AVT injection displayed the male-like olfactory investigation behavior.

## Effects of AVT on Behavior in Anurans

In contrast to the predominantly chemosensory-based salamanders, anurans depend strongly on acoustic signals to communicate. During the breeding season, male frogs and toads converge in chorusing aggregations where they signal their presence to nearby females and neighboring rival males by means of advertisement calls (56, 57). Conspecific females are attracted by these reproductive vocalizations and neighboring males typically engage in antiphonal calling contests (56, 58). Furthermore, different types of calls may be emitted depending on the behavioral context, such as aggressive calls during agonistic encounters between males or release calls emitted by males and unreceptive females to reject the amplexus of males (59). Although other sensory channels may also be employed by some species, including chemical signals and visual displays (60–62), acoustic communication is undoubtedly the leading sensory modality used by anurans in sexual and social interactions.

### Effects of AVT on Male Reproductive Behavior

**Figure 3:**
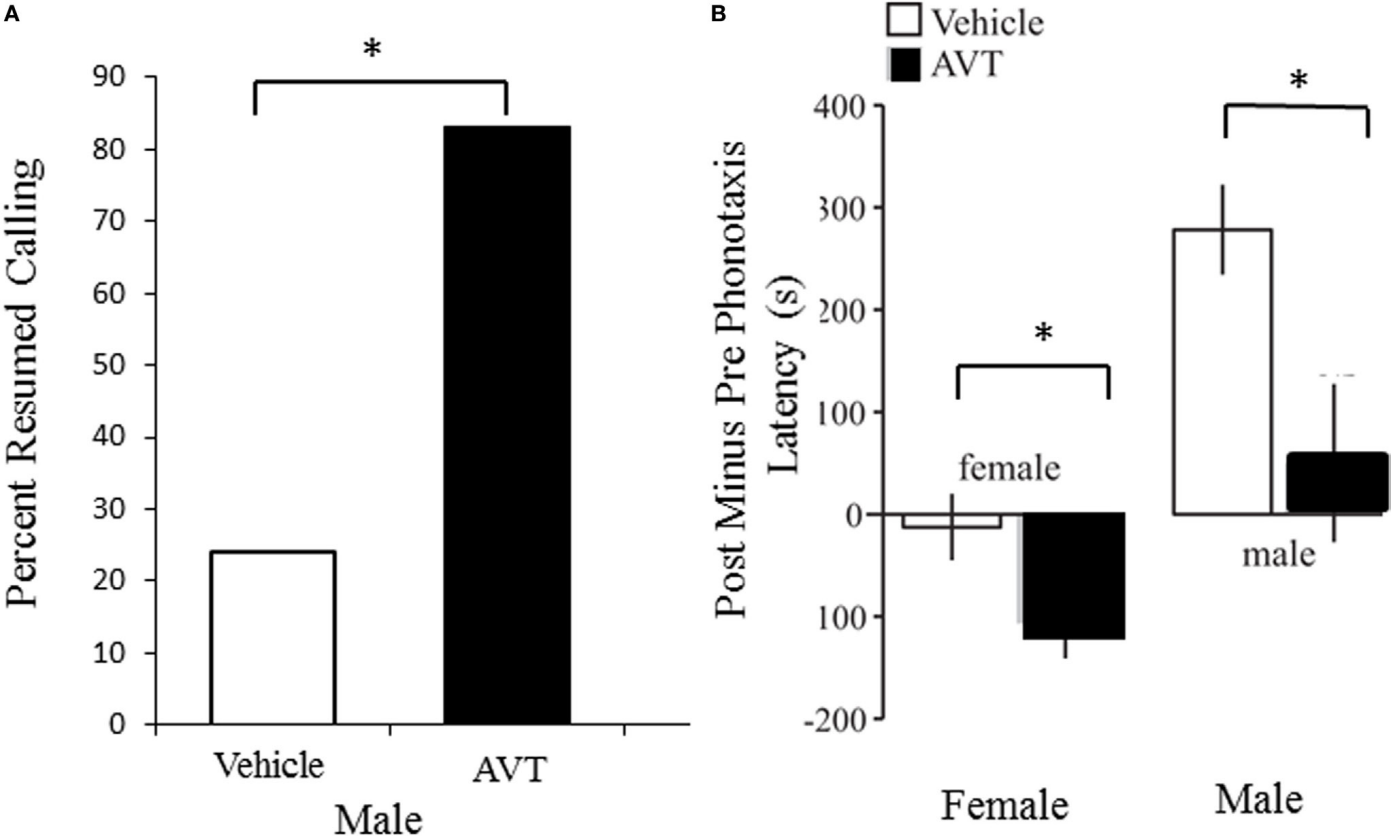
Arginine vasotocin (AVT) modulates reproductive social behavior in both male and female túngara frogs. **(A)**. Treating males with 25 mg of AVT (IP injection; *N* = 18) significantly increases the probability that males will resume calling after treatment compared to saline vehicle injection (*N* = 17). Data are from Kime et al. (69). **(B)**. Treatment with 25 mg of AVT (IP injection) decreases latency to choose and reach a speaker playing a conspecific male advertisement call in both females (left) and males (right), compared to saline vehicle injection. Data are expressed as the difference between pre and post injection latencies; *N* = 12 individuals of each sex per treatment. Note that saline injections themselves increased latency to respond in males, but AVT treatment mitigated this effect. Data are from results illustrated in Baugh and Ryan (78).

Studies conducted on salamanders have focused on a restricted number of species from the families Salamandridae and Hynobiidae (see Effects of AVT on Male Courtship Behavior). In contrast, the behavioral effects of AVT have been tested on a diversity of frog species belonging to seven different families (Table 2). Overall, studies have mainly addressed the influence of AVT on male frogs’ signaling, revealing that AVT favors the display of reproductive behaviors by promoting the emission of advertisement calls (Figure 3). Systemic AVT treatment induces three main changes in the vocal activity of male frogs, relative to control saline injections: (1) increased number of calls emitted per time unit (i.e., call rate), (2) increased likelihood to resume calling after experimental treatment (measured as the proportion of males that resumes calling after AVT injection), and (3) reduced latency to resume calling after disturbance or experimental manipulation (63–71). All three effects suggest that AVT increases the motivation to call. In addition, despite the conspicuous differences between the acoustic structure of advertisement calls emitted by different frog species, studies have shown that exogenous AVT administration modifies fine-scale temporal and spectral properties of these signals (14, 67, 69, 72–75).

**Table 2:** Effects of arginine vasotocin (AVT) on anuran social behavior.

Sex	Family	Species	Behavior	Effect of AVT	Reference
Male	Ranidae	*Rana catesbeiana* (=*Lithobates catesbianus*)	Release calling	Increase	Boyd (76)
			Advertisement calling	Increase	Boyd (38, 64)
		*Rana (*=*Lithobates) pipiens*	Release calling	Decrease	Raimondi and Diakow (77)
	Leptodactylidae	*Physalaemus (*=*Engystomops) pustulosus*	Advertisement calling	Increase	Kime et al. (69)
			Phonotaxis	Increase	Baugh and Ryan (78)
	Hylidae	*Hyla versicolor*	Advertisement calling	Increase	Semsar et al. (79), Tito et al. (72)
			Advertisement calling	No effect	Klomberg and Marler (73), Trainor et al. (74)
			Aggressive calling	No effect	Tito et al. (72)
			Release calling	No effect	Tito et al. (72)
		*Hyla cinerea*	Advertisement calling	Increase	Penna et al. (63), Burmeister et al. (68)
		*Acris crepitans*	Advertisement calling	Increase	Marler et al. (65), Chu et al. (67)
	Eleutherodactylidae	*Eleutherodactylus coqui*	Advertisement calling	Increase	Ten Eyck (80)
			Aggressive calling	Increase	Ten Eyck and ul Haq (70)
			Parental care	No effect	Ten Eyck and ul Haq (70)
	Bufonidae	*Bufo (*=*Anaxyrus) cognatus*	Advertisement calling	Increase	Propper and Dixon (66)
			Amplexus	No effect	Propper and Dixon (66)
	Dendrobatidae	*Ranitomeya imitator*	Parental care	Decrease	Schulte and Summers (81)
	Pipidae	*Xenopus tropicalis*	Advertisement calling	Increase	Miranda et al. (71)
			Amplexus	No effect	Miranda et al. (71)

Female	Ranidae	*R. catesbiana (*=*Lithobates catesbeianus)*	Release calling	Decrease	Boyd (76)
			Phonotaxis	Increase	Boyd (38, 64)
		*Rana (*=*Lithobates) pipiens*	Release calling	Decrease	Diakow (82), Diakow and Nemiroff (48), Raimondi and Diakow (77), Raimondi and Diakow (77)
	Leptodactylidae	*Physalaemus (*=*Engystomops) pustulosus*	Phonotaxis	Increase	Baugh and Ryan (78)
	Dendrobatidae	*R. imitator*	Parental care	No effect	Schulte and Summers (81)

*For those species in which vocal behavior was studied (i.e., advertisement, aggressive, and release calling), “Increase” refers to an augmentation of the probability of calling or calling rate, or a reduction in latency to call (see text for further descriptions). Studies that measured any of these calling parameters but did not find an effect of AVT are labeled as “No effect.” For the studies evaluating the effects of AVT on phonotaxis, “Increase” refers to an increase in sexual arousal, measured as a shorter latency to approach a speaker playing advertisement calls, or increased choice behavior*.

Although evidence for AVT-mediated modifications of the vocal behavior of male frogs is compelling, the significance of these alterations for intra- and inter-sexual communication is poorly understood. Experiments conducted on gray treefrogs (*Hyla versicolor*) have provided valuable insights into the influence of AVT on male–male social interactions. In this species, AVT increases both the number of pulses and the duration of male advertisement calls (73). Also, AVT-injected males placed in the proximity (within 10 cm) of another conspecific calling male are more likely to emit advertisement vocalizations and, furthermore, take over the calling site of the resident male without engaging in physical aggression (79). This is an uncommon outcome, as in other frog species resident males usually retain calling sites when confronted by intruders (83, 84), suggesting that AVT may either increase the aggressiveness of *H. versicolor* advertisement calls or, alternatively, make them more competitive by improving their attractiveness (73). Intriguingly, AVT-induced changes in the calls of male gray treefrogs occur only in the presence of other nearby calling males, as these modifications are not evident when males are placed farther than 2 m away from other vocally active males (74). Altogether, these results indicate that the influence of AVT on male frogs’ reproductive behavior are relevant for intra-sexual communication, and, most notably, that AVT-induced modifications of male signaling may be shaped by the surrounding social (acoustic) context. Similar conclusions can be derived from experiments conducted on male cricket frogs (*A. crepitans*) (67). The significance of the social context highlighted by these studies pinpoints a fundamental issue to be considered in future studies. Research aimed at evaluating the effects of AVT on frogs’ reproductive behavior should provide a careful characterization of the acoustic environment in which experiments are conducted. In addition, the acoustic context could be homogenized by using playback designs [e.g., Ref. (69)].

Arginine vasotocin-induced changes on male calls can also have implications for female responses, an issue that has been considered central to elucidate the relevance of AVT modulation on anuran reproductive behavior (85). Some studies have found that AVT modifies call properties likely to be relevant for female choice (65, 73); nevertheless, female preference for AVT-modified male calling has only been tested in túngara frogs [*Physalaemus (*=*Engystomops) pustulosus*]. The advertisement calls emitted by male túngara frogs consist of a frequency-modulated note called a “whine,” which can be followed by one or more secondary notes termed “chucks.” Males emit shorter whines with higher initial frequency and add more chucks to their calls following AVT administration. As females exhibit a strong preference for calls having chucks (86, 87), Kime et al. (69) initially hypothesized that AVT improves the attractiveness of male vocalizations. To evaluate this possibility, Kime et al. (75) conducted two-speaker female phonotaxis trials with natural male calls emitted before and after AVT treatment. These experiments showed that females consistently preferred whines (chucks were initially cutoff for these female choice experiments) emitted before males were administered AVT. When females were tested with complete calls (i.e., whines including subsequent chucks), there was no difference in female preference between calls emitted before and after AVT injection. Together these results indicate that AVT renders the obligatory part of the male advertisement call (the whine) unattractive to females, and that call attractiveness is at least not enhanced by AVT when attractive notes (chucks) are added to the vocalizations. Because of the few studies on female responses to male AVT-modified calls it is not clear whether social context or female reproductive state would change the results. However, given that male responses appear to be context dependent, the environmental settings in which experiments with females are conducted should certainly be considered.

An alternative approach to treatment with exogenous AVT is to assess the relationship between brain AVT levels and behavioral state. AVT cell populations are widespread in the anuran brain [see AVT in Anurans (Frogs and Toads)], and AVT expression levels in one such nucleus has been related to the display of male reproductive behaviors. Silent satellite cricket frogs (*A. crepitans*) present more AVT staining in the nucleus NAcc relative to sexually active males (i.e., calling individuals) (14). This forebrain region is thought to be involved in weighting the salience of social stimuli across vertebrates (88). As AVT administration increases the motivation to call in this species (65, 67), the authors suggested that the emission of vocalizations is associated with an increase of brain AVT release (14), thus resulting in a decreased level of observed NAcc AVT due to depletion by this increased secretion. In consonance with this interpretation, early in the breeding season of the frog *Hyla cinerea*, when the vocal activity of males is typically high, AVT-ir cells in the NAcc of males are smaller and less abundant relative to males measured once the mating season is over (15). Neither study found evidence of an association between calling activity and AVT cell number or size in other brain nuclei. It is possible that some other metric of brain AVT level or activity [e.g., high performance liquid chromatography with fluorescence detection as has been applied to measure fish brain AVT levels (89, 90)], would reveal relationships with calling in other areas. However, it should be noted that AVT/AVP has multiple effects on social behavior across vertebrates beyond social signaling, as well as effects on physiological processes. It is possible that these other frog AVT centers are associated with one or more of those other functions, as Kabelik et al. (24) found in *Anolis* lizards (see Effects of Vasotocin on Behavior in Reptiles).

There is evidence that AVT may modulate other aspects of male amphibian reproductive behavior beyond signal production. For example, some frogs rely on acoustic cues (e.g., male advertisement calls) to orient toward breeding aggregations (91), and a recent study reported that the phonotactic responses of male túngara frogs to conspecific advertisement calls is increased by AVT administration (78), highlighting the role of this peptide in the recruitment of males to mating assemblages. Studies have also evaluated the effect of AVT on the amplectic clasping of male frogs. Similarly to the courtship behavior of rough-skinned newts, male frogs embrace females for effective mating and receptive females subsequently release their eggs, which are externally fertilized by males’ sperm (57). Despite the behavioral similarities between frog and *T. granulosa* amplexus, differences have been found in AVT’s modulation of clasping behavior among these amphibians. While AVT strongly induces amplectic behavior in *T. granulosa* (45), experiments conducted with western clawed frogs (*Xenopus tropicalis*) and great plain toads [*Bufo* (=*Anaxyrus*) *cognatus*] have failed to elicit amplexus through systemic AVT treatment (66, 71). Still, the influence of AVT on frog amplectic behavior cannot be dismissed, as male *X. tropicalis* attempt to clasp females more often after being injected with a combination of human chorionic gonadotropin (hCG) plus AVT relative to frogs receiving hCG only (71).

### Effects of AVT on Male Aggression

In general, agonistic encounters among male frogs occur when they defend calling territories from other neighboring males. These aggressive interactions are usually solved by means of the emission of aggressive vocalizations (59), although conflicts may escalate to physical aggression in some species [e.g., Ref. (92)]. In frogs, agonistic vocal interactions are generally modulated by androgens and corticosterone (93, 94), but AVT is also important. However, the influence of this neuropeptide on anuran aggression is not as clear as its influence on the emission of advertisement signals.

One species where this was examined is the cricket frog (*A. crepitans*), where males have a “graded communication” system (57, 59, 95, 96) in which they modify their pulsed advertisement call in a graded fashion in order to signal increasing levels of aggressiveness to challenging conspecific calls rather than switch to a different aggressive call (97–101). Relative to normal advertisement calls, aggressive-like vocalizations include a combination of temporal (i.e., increased call duration and number of pulses, among others) and spectral (i.e., lower dominant frequency) modifications (98, 102). By means of a set of field experiments and detailed acoustic analyses, Marler et al. (65) demonstrated that treatment with AVT increases the probability of calling and also induces call modifications typical of less aggressive males when experimental subjects are stimulated with the natural surrounding chorus. A later study suggested that the apparent reduction in aggression levels found by Marler et al. is probably a by-product of an increased motivation to call (67). Whether or not this is the case, the calls emitted by AVT-injected cricket frogs are likely to be perceived as less aggressive variants by other neighboring conspecifics. In contrast, a study with male *H. versicolor*, which have separate advertisement and aggressive calls rather than using a graded call system, failed to find changes in the emission of aggressive calls after AVT treatment (72). Furthermore, different effects of AVT have been described for Puerto Rican coquí frogs (*Eleutherodactylus coqui*). After mating, male coquí frogs stay near the egg clutch providing parental care, a period during which their vocal activity is typically low (103). Both male and female coquí frogs emit distinct aggressive calls in response to the intrusion of other conspecifics into their shelters (104). In paternal *E. coqui*, the probability of emitting aggressive calls is increased following AVT administration (70). In addition, AVT treatment stimulates the emission of advertisement vocalizations in non-calling satellite male *E. coqui* (80).

These studies reinforce the notion that the effects of AVT on anuran behavior are species- and context-dependent, and, in the case of coquí frogs, dependent on the reproductive status of the males. The mechanisms responsible for such dependency are unknown, but one possibility is that the behavioral effects of AVT depend on the overall hormonal states associated with different behaviors. Differences in androgen and corticosterone levels have been reported between calling and silent male frogs (105, 106) and paternal *E. coqui* have lower circulating testosterone levels than calling males (107). This is a promising avenue for future research, as the behavioral effects of AVT have been shown to be dependent on the administration of androgens (63). Moreover, gonadal steroids (38, 108) and melatonin (16, 17) have been shown to modulate the expression of AVT and its putative receptor in brain areas. These effects may be part of the mechanism by which these other hormones influence frog social behavior.

### Effects of AVT on Female Reproductive Behavior

Arginine vasotocin modifies female reproductive behavior as well as male calling (Table 2; Figure 3). For instance, female bullfrogs and túngara frogs approach a loudspeaker playing male calls faster following AVT treatment (64, 78). Whether AVT affects females’ responsiveness to different call variants, such as attractive and unattractive vocalizations, is unknown.

Calling behavior, including the production of female advertisement calls, is not absent in female frogs, and it has been described in numerous species belonging to over 10 families (33, 109, 110). AVT’s effect on natural female advertisement vocalizations has not been tested. However, an interesting set of experiments indicates that AVT does have the ability to unlock female calling. Normally, female *H. cinerea* frogs do not emit calls; however, hormonal manipulations have successfully elicited vocalizations in this species. AVT promotes the emission of calls in testosterone-implanted female *H. cinerea* stimulated with conspecific calls, while non-implanted intact females remain silent. In contrast, saline injections do not evoke vocalizations in intact and testosterone-implanted females (63). The spectral and temporal characteristics of female vocalizations were similar to the advertisement calls emitted by intact males, except for the low frequency peak, which was about 350 Hz higher in females. These results demonstrate that testosterone and AVT act synergistically to induce mating-like vocalizations in female *H. cinerea*. This is a puzzling result, as reproductive vocalizations have not been reported for female Hylids [see Ref. (111) for an updated survey of female frogs’ vocalizations], yet the circuitry for calling is apparently present and modifiable by AVT.

One type of social signal produced by females as well as by males in many frog species is the “release call” (57). Release calls are produced by vibrating trunk muscles in response to another individual’s grasping, as males normally do with females during amplexus. This display can have both acoustic and vibratory components as is the case in some Bufonidae species (112, 113), or only trunk vibrations [e.g., *Pleurodema thaul* (114)]. The release signal communicates a lack of receptivity (in females) or inappropriate clasping (in males), and hence it signals the amplexing individual to release the signaler. The first study of AVT effects on anuran social signaling was in fact done on release calls. Diakow (82) reported that a systemic AVT injection reduced release calling in female bullfrogs [*R. catesbeiana (*=*Lithobates catesbeianus*)], which would mean that AVT increases receptivity. Boyd (76) confirmed the female effect in leopard frogs [*Rana (*=*Lithobates) pipiens*]. These effects on release calling are consistent with AVT’s enhancement of female phonotaxis to male advertisement calls (64, 78).

Arginine vasotocin modulation of male release calling is inconsistent and difficult to interpret, as this neuropeptide has been reported to decrease release calling in both male and female leopard frogs (77), but increases release calling in male bullfrogs while it decreases this response in females (76). Furthermore, these changes occurred only in the spring; AVT treatment in the fall had no effect on either sex. In addition AVT has no effect on release calling in male *H. versicolor* other than to decrease the duration of individual release calls (72). The significance of these changes for natural male sexual behavior are not clear, as a decrease in male release calling apparently lacks an adaptive role, and it is not known how male reproductive state correlates with natural release calling. Overall it is difficult to interpret the significance of the opposite effects that AVT has on release calling in closely related Ranid species, or even if the experimentally induced change in release calling is an epiphenomenon of AVT effects on calling, social motivation, or systemic physiological regulation.

### Unanswered Questions

Research evaluating the influence of AVT on anuran behavior has largely focused on male behavior, particularly on the emission of acoustic signals under various social contexts. These studies prompt two general conclusions: (i) AVT promotes the emission of reproductive signals and (ii) the modulation of male sexual and aggressive behaviors by AVT is dependent on the social context. All of this work has focused on acoustic communication. The influence of AVT on the emission of non-acoustic signals now arises as an important question for future research, as a recent study demonstrated that the emission of agonistic visual displays (foot-flagging) is modulated by androgens in Bornean rock frogs (*Staurois parvus*) (115). Also, male dwarf African clawed frogs (*Hymenochirus* sp.) have breeding glands that produce odorants effective for female attraction (116), but influences of androgens or peptides on such secretions have not been explored.

Male-biased AVT research is intriguing but female behavior has been neglected. Female frogs also display many of the behaviors that have been studied in males, including aggression (117) and even the emission of different kinds of vocalizations (33). None have been examined for AVT modulation. AVT effects on female phonotaxis, the most conspicuous reproductive behavior of this sex, have been rarely studied until recently (78). One additional social behavior that females and males display in some species is parental care (81, 118). Shulte and Summers (81) recent study indicates that AVT might influence some aspects of egg-care behavior, although decreasing some aspects while increasing others. The more complex social behaviors seen in some frog species deserve more attention to determine if the conserved functions of the nonapeptides extend to these types of amphibian behaviors.

Finally, although the behavioral effects of AVT are clearly documented, what remains unclear is what neural mechanism might account for these changes. In urodeles, AVT research has extended to studies of its influence on modification of the elemental sensory and motor components of social communication systems in this group (see Effects of AVT on Male Courtship Behavior). That might explain at least some of the facilitation of this nonapeptide on urodele social behavior. This type of research is almost wholly lacking in anurans (and reptiles). The one exception is early work by Penna et al. (63) that examined midbrain auditory thresholds after manipulations of both testosterone and AVT. They found AVT reduced midbrain sensitivity to mid- and high-frequency sound, but only in males, following castration with or without testosterone implant. The authors noted that this was puzzling, given that the same treatment stimulated calling in the testosterone-implanted males. This is only one paper and, therefore, only begins to address how AVT is modifying neural systems, but it does at least indicate that changes on the sensory side of the communication system may be induced by AVT, and suggests that further work like this is warranted, perhaps modeled on the neurophysiological experiments on sensory and sensory–motor centers done in urodeles.

## Effects of Vasotocin on Behavior in Reptiles

Compared to work in amphibians and other vertebrates, there have been relatively few studies in reptiles directly linking AVT to behavior. This is true despite the long history of research on lizard aggression, male displays, and social hierarchy formation (119–121), all of which are prime targets of AVT/AVP research in other vertebrates. What research has been done focuses mainly on social behavior (aggression and to a lesser degree courtship) rather than on social signal production *per se*. Although few in number, behavioral endocrinology studies of reptile AVT shows that the general function of this nonapeptide in modulating male aggression is conserved in reptiles. As in amphibians, the effects of AVT in lizards are complex and context dependent.

The majority of research that links brain AVT function with aggression or other social behaviors in reptiles has taken the indirect approach of relating AVT cell number or staining density to individual or group behavioral profiles. Within-sex differences indirectly implicate brain AVT in social regulation. Male green anoles held in laboratory conditions will, when paired, interact with aggressive displays and ultimately form stable dominant/subordinate dyads (121–123). Hattori and Wilczynski (23) found that after 10 days of stable paired housing, dominant males had a higher number of AVT cells in the POA than did subordinate males, with no difference apparent in other AVT populations. Although not specified in this publication, the area affected most likely includes, but may not be limited to, cell population equivalent to the PVN of mammals and reptiles. Singly housed control males had POA AVT cell counts intermediate between dominants and subordinates, a pattern that suggests a rise in POA AVT in dominants and a decline in subordinates as a function of this ongoing difference in social state.

Kabelik et al. (24) used a more sophisticated double labeling immunocytochemistry approach to identify the participation of various AVT populations in different types of social behavior in brown anole lizards (*Anolis sagrei*). They double-labeled neurons for AVT and the immediate early gene *Fos*, a marker of neural activity, in males engaged in aggressive or sexual interactions and found differential activation of brain AVT populations. Paraventricular nucleus (PVN) AVT cells were active during aggressive encounters with another male, with a positive correlation between aggression levels and *Fos* activation. In contrast, AVT cells in the POA were preferentially active during sexual interactions (courtship and copulation) with a female. SON and BNST AVT cells were active in both social situations. The relationship between the activation results of Kabelik et al. and the state-dependent differences reported by Hattori and Wilczynski have not been reconciled. One might have hypothesized a greater dominant/subordinate activity difference in the aggression-activated PVN AVT cells, given that differences in aggressive behavior and displays are the preeminent causes and consequences of social status differences in anoles (121, 123–125). However, as is the case in many vertebrates, dominant male anoles differ in many behavioral and physiological traits beyond aggression (121, 124, 126), any of which could be related to AVT changes in different nuclei. Furthermore, a slowly emerging, stable state difference in the brain following long term experience in a social status is a different characteristic than immediate activation during a specific behavior. Although much work remains to be done in order to understand the role of various AVT populations in social behavior and their modification as a consequence of that behavior, the results of these studies suggest a complex network of forebrain AVT cell populations participating in a variety of male social behaviors.

**Figure 4:**
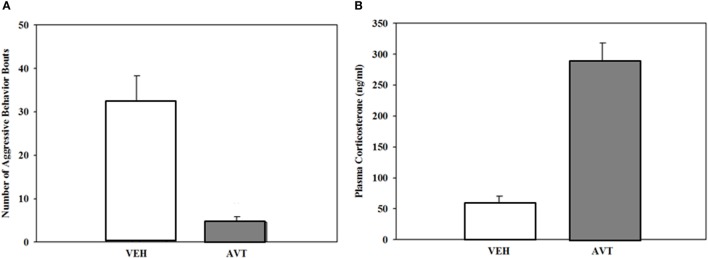
**(A)**. Reduction in aggressive responses (mean + SEM) to a mirror in male green anoles following intraperitoneal injection of arginine vasotocin (AVT) (15 µg AVT/50 μl reptile Ringer; *N* = 10) or vehicle (*N* = 6). **(B)**. Increase in plasma corticosterone in the same animals following treatment (mean + SEM). From data in Dunham and Wilczynski (127).

Few studies have taken the more direct approach by treating males with exogenous AVT. Dunham and Wilczynski (127) did this using intraperitoneal injections of AVT in male green anoles. AVT injections decreased, rather than increased, aggressive displays to a mirror (Figure 4). Although at first this may seem contrary to expectations, in fact AVT has been found to have opposite effects depending on species and context. Green anoles are territorial, and most consistently across species of birds and fish AVT tends to suppress aggressive behavior in territorial individuals, species, or morphs while stimulating it in non-territorial or socially gregarious animals (4, 128). AVT did not, however, influence aggression or the outcome when AVT treated male anoles interacted with size-matched saline injected males. This is of course a more complicated situation where the interaction between the two males determines the level of behavior in each as well as the outcome of the encounter. AVT treatment effects put the increased POA AVT cell number in dominant males into perspective. This dominant-subordinate difference may represent lower release of the aggression-suppressing peptide in the more aggressive dominants. Note that this is similar to the argument regarding AVT cell variation in male cricket frogs, that is, that the higher release of the call-stimulating peptide results in smaller sized and a lower number of AVT-containing cells in calling vs. satellite males (14).

Interestingly, AVT treatment of male anoles does not change the number of courtship displays to a novel female; however, females were significantly more responsive to AVT-injected males (127). AVT must have modified some aspect of male courtship signaling, either through subtle changes in the form of the observable visual displays not yet documented, or through some modulation of signals not readily apparent such as pheromonal signals. Chemical signaling is poorly understood in anoles but is present in many lizards (129). AVT does influence responses to conspecific odors in male European Common Lizards (*Zootoca vivipara*) (130), where it suppresses attraction to odors of other conspecific males. The effect is restricted to smaller males, highlighting again the complex context and class dependence of AVT’s effects. Whether females are similarly affected by AVT is unknown.

Social bonding, which is a function of nonapeptides in mammals (1), is usually considered beyond the realm of reptiles, and in fact most lizards, turtles, and snakes do not pair-bond or show parental care. Some viviparous snakes do, however, show mother–offspring brooding-like behavior and defense of their offspring. Pigmy rattlesnakes (*Sistrurus miliarius*) are an example, and a recent paper (131) reported that blocking AVT/AVP V1a receptors eliminated mothers’ preferences for aggregating with their offspring. It is worth noting that in addition to many species of snakes, skinks in the order Scincidae also show parental care, and crocodilians are virtually bird-like in their nesting behavior. This species diversity provides ample opportunities for extending the investigation of reptilian AVT into areas of social behavior beyond simple aggression and courtship responses, just as the expected diversity of frog social behavior beyond male advertisement calls and female responses does for amphibians.

## Peripheral Effects of Vastocin in Amphibians and Reptiles

In addition to work on AVT’s behavioral effects, a great deal of research has been done on the peripheral physiological and endocrinological functions of this peptide in both amphibians and reptiles. In reptiles, AVT, like AVP in mammals, is a peripherally acting peptide hormone influencing osmoregulation, which in reptiles is also tied to thermoregulation, and stimulating smooth muscle contraction, including oviduct contractions associated with oviposition (55, 132–136). Similarly, osmoregulation is an important peripheral function of amphibian AVT (137, 138). Frogs “drink” water through their ventral skin, which is the main way in which they stay hydrated. Treatments with exogenous AVT stimulate water absorption in both anurans (138) and urodeles (139). Frog ventral skin expresses AVT receptors (140) verifying the peripheral action of AVT there. ICV AVT injections also stimulate water absorption (141) indicating that AVT also acts as a central nervous system modulator of hypothalamic centers regulating water retention. Given the importance of water for survival and egg laying in amphibians, it is possible that AVT’s osmoregulatory effects may have an unrealized influence on their behavior. Diakow and Nemiroff (48) in fact suggested that the AVT triggered decrease in female release calling was due to water absorption, leading to abdominal extension mimicking a large egg mass. This may have contributed to her experimental results, but it is not at all clear how this would account for the enhancing effects of AVT on release calling in males.

**Figure 5:**
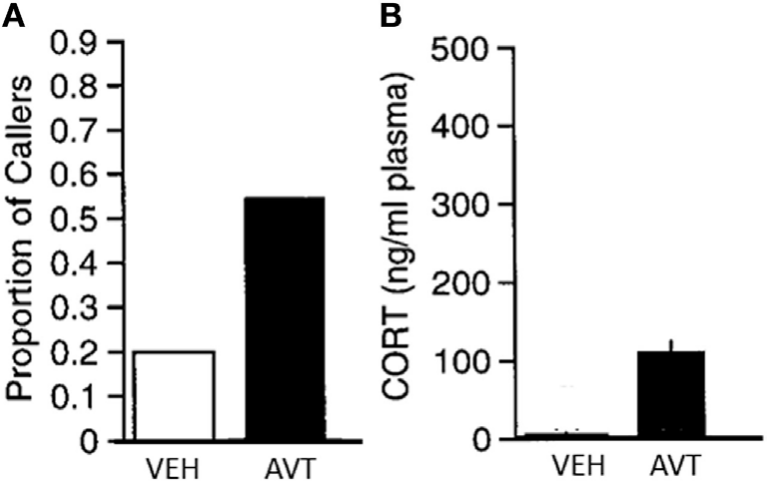
**(A)** Significant increase in the proportion of male green treefrogs that resumed spontaneous calling following and IP injection of 20 μg arginine vasotocin (AVT) compared to vehicle (saline) injection. **(B)** Significant increase in plasma corticosterone (mean + SEM) following injection with 20 μg AVT compared to vehicle (saline) injection. Results show that AVT increases calling even when stimulating corticosteroid release. *N* = 10 males per group. From data in Burmeister et al. (68).

Particularly important in the context of its effects on social behavior, AVT is a potent stimulator of adrenal gland steroid hormone secretion in both amphibians (Figure 5) and reptiles (68, 127, 142), just as AVP is in mammals. High cortisol levels can dramatically change social behavior in many vertebrates by various mechanisms including interaction with other hormones and, although less well studied, possibly through glucocorticoid effects on glucose metabolism and related energetic functions. Although peripheral AVT action is less a concern in studies investigating natural variation of brain AVT measures with behavior, those peripheral effects are problematic for experiments employing systemic treatment with exogenous AVT or vasopressin receptor blockers, as these procedures will influence bodily physiological functions in addition to, and perhaps more than, AVT signaling in the brain. In some cases, such as the decrease in aggression seen in anoles after systemic AVT treatment (127), it is possible that the coincident rise in corticosteroids might contribute to the AVT’s effect in decreasing aggression. On the other hand, the fact that AVT treatment in frogs increases calling rates even though it also increases corticosteroids (68) (Figure 5) suggests that AVT positively influences calling independent of, and in spite of, any negative effects of elevated corticosteroids. Understanding the role of AVT’s peripheral endocrinological and physiological effects on social behavior remains a critical, but largely overlooked, component of AVT’s behavioral endocrinology.

## Summary and Discussion

Arginine vasotocin’s anatomy and function in amphibians and reptiles are similar to that of AVP in mammals, both in their general features and in the questions they raise. AVT-containing cells, consisting of both magnocellular and parvocellular neurons, are found mainly in limbic forebrain and hypothalamic areas with a widespread fiber distribution throughout the central nervous system marked by a particularly heavy innervation of the limbic Social Decision Network and associated social communication areas of the brain. There is generally a male-biased sexual dimorphism in the anatomy, although with several reported exceptions. Finally, AVT has both peripheral physiological effects common across vertebrates and modulatory influences on social behavior and communication. These behavioral effects are most consistently observed in males, but are now seen as modulating female social behavior as well in the few cases where it has been investigated. Within this general framework, significant diversity within and across species is apparent, leading to the first and most fundamental question regarding AVT’s (and AVP’s) modulation of social behavior: what is the mechanism by which this important neuropeptide exerts its effects?

Various studies on amphibians and reptiles point to multiple points at which AVT could influence an organism’s responses to, and production, of social signals as well as modulate other aspects of socially cued behavior. There is evidence that AVT modifies chemical, visual, and auditory processing in urodeles and anurans (39, 63), channels used in various species for their communication. There is also direct physiological evidence as well as suggestive neuroanatomical data that implicate action on motor areas (40). Moreover, the cellular location and dense AVT innervation of the NAcc points to an impact on signal salience or social reward, and more broadly the presence of AVT cells or fibers in multiple areas of the Social Decision Network and on limbic pallial/cortical areas suggests an impact on motivational and higher cognitive functions. Furthermore, AVT’s influence on peripheral physiology and endocrinology is significant; how this does or does not account for any of the experimental results of AVT treatment remains an important issue to address. Of course, it is possible that AVT acts in multiple, and perhaps independent, ways, both centrally and peripherally, or that AVT in some way acts to bind these areas together by modifying a functional network linking them. An important step toward understanding exactly what this peptide is doing would be formulating truly testable hypotheses explaining the mechanisms by which changes in peptide levels result in changes in specific aspects of social behavior.

The diversity seen in the AVT system represents both a challenge to understanding its mechanism of action and a potential tool to investigate it. Although the widespread fiber distribution is common across species, species variation in the location of AVT cells themselves is apparent in Table 1. This diversity needs to be viewed cautiously, as reports of AVT cell location in urodeles, anurans, and reptiles, go back over 30 years, and systematic, well controlled comparative studies remain to be done with modern methods. Nevertheless, if variation in the anatomical distribution of AVT neurons is real, an analysis of whether or not this is functionally relevant could make an important contribution to understanding the structure-function relationship of AVT and other neuroactive peptides.

Explaining the diversity of AVT’s effects remains a major challenge as well as an opportunity to delve deeper into the mechanisms by which this peptide operates. The AVT system is a dynamic one, sensitive to multiple factors within and across individuals. In addition to a consistent sex difference within species, AVT characteristics vary within individuals based on seasonal, social, and hormonal state. The influence of steroid hormones on AVT levels and possibly action is particularly profound. This diversity has already been used to confirm AVT’s modulation of various social behaviors, and this provides a foundation for deeper explorations of AVT’s mechanisms. The influence of melatonin on AVT neurons shown in anurans (16) is also an aspect that deserves further explorations in the other groups. The variation in AVT’s influence across species is also significant. Whereas AVT consistently influences social communication and aggression across species, what is influenced and how it is modulated can vary greatly. For example, AVT influences chemical signaling in urodeles, vocal signaling in anurans, and visual displays in reptiles; in the first two taxa, AVT increases the propensity to signal, whereas in reptiles, at least in an aggressive context, it decreases visual signaling (spontaneous display behavior has not been assessed in lizards). Moreover, all possible effects of AVT on male frog aggressive calling have been reported: decreased aggressiveness (65), increased aggressiveness (70) and no effect (72). Why this is so remains an enigma; however, the species- and context-dependence of AVT’s effects on aggression is a phenomenon observed in other taxa as well. How the link between AVT function and behavioral output can vary so dramatically remains an important gap in our knowledge. Although a challenging issue, the species diversity across urodeles, anurans, and reptiles in social behavior also represents an opportunity to dissect the way in which AVT acts on the neural systems responsible for social behavior. Anuran species, for example, range in calling sex dimorphism from vocal advertisement signaling being strictly a male behavior, to females producing audible calls other than advertisement calls, to both males and females producing advertisement calls. Visual displays are an important adjunct to vocal signals in some anuran species, but not others. Release signals produced by both males and females have both audible and vibratory components in some species, but only vibratory in others. How is this diversity within and between sexes reflected in either the anatomical or physiological characteristics of brain AVT? Extending the type of structure-function analysis used to implicate AVT function within species to the diversity seen among species could provide real insight into how social peptides evolve and operate.

## Author Contributions

All authors discussed and planned the paper. Each author was the primary author for approximately 25% of the paper. All authors reviewed and edited all sections of the paper, which was then compiled by the corresponding author.

## Conflict of Interest Statement

The authors declare that the research was conducted in the absence of any commercial or financial relationships that could be construed as a potential conflict of interest.

## Abbreviations

III, third ventricle; AH, anterior hypothalamus; BNST, bed nucleus of the stria terminalis; Dp, dorsal pallium; Lp, lateral pallium; Ls, lateral septum; MA, medial amygdala; Mp, medial pallium; Ms, medial septum; NAcc, nucleus accumbens; PAG, periaquaductal gray; POA, preoptic area; PVN, paraventricular nucleus; SCN, suprachiasmatic nucleus; SON, supraoptic nucleus; Str, striatum; Tel, telencephalon; Th, thalamus; VL, ventrolateral hypothalamus; VM, ventromedial hypothalamus.
